# Long-Lasting, Antinociceptive Effects of pH-Sensitive Niosomes Loaded with Ibuprofen in Acute and Chronic Models of Pain

**DOI:** 10.3390/pharmaceutics11020062

**Published:** 2019-02-01

**Authors:** Francesca Marzoli, Carlotta Marianecci, Federica Rinaldi, Daniele Passeri, Marco Rossi, Paola Minosi, Maria Carafa, Stefano Pieretti

**Affiliations:** 1 Istituto Superiore di Sanità, National Center for Drug Research and Evaluation, 00161 Rome, Italy; francesca.marzoli@iss.it (F.M.); paola.minosi@iss.it (P.M.); 2 Department of Drug Chemistry and Technology, Sapienza University of Rome, 00185 Rome, Italy; carlotta.marianecci@uniroma1.it (C.M.); maria.carafa@uniroma1.it (M.C.); 3 Center for Life Nano Science@Sapienza, Istituto Italiano di Tecnologia (ITT), 00161 Rome, Italy; federica.rinaldi@uniroma1.it; 4 Department of Basic and Applied Sciences for Engineering, Sapienza University of Rome, 00161 Rome, Italy; daniele.passeri@uniroma1.it (D.P.); marco.rossi@uniroma1.it (M.R.); 5 Research Center for Nanotechnology Applied to Engineering, Sapienza University of Rome (CNIS), 00185 Rome, Italy

**Keywords:** Ibuprofen, pH-sensitive niosomes, Pain, Analgesia, NSAIDs

## Abstract

Ibuprofen is one of the non-steroidal anti-inflammatory drugs (NSAIDs) widely used to treat pain conditions. NSAIDs encounter several obstacles to passing across biological membranes. To overcome these constraints, we decided to study the effects of a new pH-sensitive formulation of niosomes containing Polysorbate 20 derivatized by Glycine and loaded with ibuprofen (NioIbu) in several animal models of pain in mice. We performed two tests commonly used to study acute antinociceptive activity, namely the writhing test and the capsaicin test. Our results demonstrated that NioIbu, administered 2 h before testing, reduced nociception, whereas the free form of ibuprofen was ineffective. In a model of inflammatory pain, hyperalgesia induced by zymosan, NioIbu induced a long-lasting reduction in hyperalgesia in treated mice. In a model of neuropathic pain induced by sciatic nerve chronic constriction, NioIbu reduced both neuropathy-induced allodynia and hyperalgesia. The results obtained in our experiments suggest that pH-sensitive niosomes containing Polysorbate 20 derivatized by Glycine is an effective model for NSAIDs delivery, providing durable antinociceptive effects and reducing the incidence of side effects.

## 1. Introduction

The non-steroidal anti-inflammatory drug (NSAID) Ibuprofen (α-methyl-4-(2-methylpropyl) benzeneacetic acid, IBU) is commonly used in the treatment of pain, fever, and inflammatory diseases. Ibuprofen was discovered in 1960 for the treatment of some pain conditions and inflammatory autoimmune diseases, such as rheumatoid arthritis [1]. In 1961, Ibuprofen became available in tablet form, and in 1983 was launched as a topical formulation. Ibuprofen is an analgesic drug that inhibits the production of cyclooxygenase (Cox-2) pathway-derived prostaglandins, which increase in inflamed tissues and control inflammatory disorder [2,3]. As is well-known, prostanoids mediate the sensation of peripheral and central pain, increasing membrane excitability and reducing the threshold of nociceptor stimulation [4].

While ibuprofen is completely absorbed after oral administration [5], the plasma level is still low several hours after topical application [6]. Various studies have reported different techniques to increase the permeability of cell membranes and the transdermal transport of ibuprofen and other drugs: preparing colloidal microstructures with lysinate and lecithin [7,8], nanoscaled emulsion with palm olein esters [9], iontophoresis [10], and using surfactants and various vehicles, such as the niosomes [11,12]. Niosomes are unilamellar or multilamellar non-ionic surfactant vesicles similar to liposomes that can be used as therapeutic nanocarriers. The main characteristic of these vesicles is their capability to encapsulate both hydrophilic and lipophilic drugs. Hydrophilic drugs are encapsulated in the inner core where the aqueous compartment is located, while lipophilic drugs are encapsulated into the lipophilic domain of the bilayer [13,14,15].

**Figure 1:**
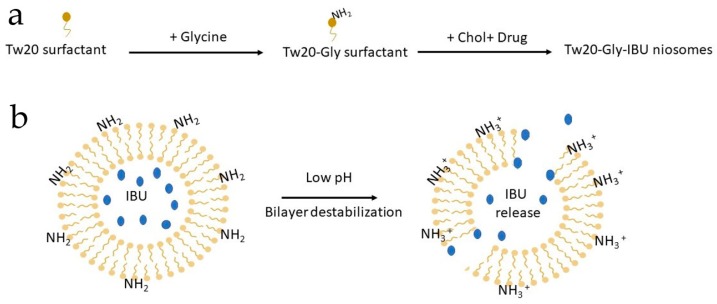
Cartoon describing: (**a**) niosomal preparation by a derivatized surfactant; and (**b**) bilayer destabilization at an acidic pH and consequent drug release. IBU is for Ibuprofen.

The basic components of niosomes are non-ionic surfactants in addition to cholesterol (Chol). The surfactants are mainly composed of two distinct regions and are classified as anionic, cationic, amphoteric, and non-ionic according to the feature of the hydrophilic head, which contains sulfonate or ammonium salts of fatty and zwitterionic acids. Non-ionic surfactants have no charge groups in their hydrophilic heads and are able to form niosomal vesicles that represent an innovative system with better performance compared to conventional drug delivery systems. Indeed, niosomes are more stable than liposomes. They show greater bioavailability compared with conventional dosage forms because they are able to increase the permeation of drugs through the skin [16]. Furthermore, they can be employed for oral and parenteral administration, protecting drugs from biological enzymes and the environment. Moreover, the surfactants employed in the preparation of niosomes are less expensive and more versatile than the phospholipids used for liposomal formulations [17]. To obtain a site-specific drug release, pH-sensitive molecules can be added to the formulation or used to derivatize the surfactants employed. It is well known that some pathological states are associated with pH profiles different from that of normal tissues. Examples include ischemia, infection, inflammation, and cancer, which are often associated with acidosis. Extracellular pH values ranging from 5.5 to 7.0 have been detected in inflamed tissues associated with bacterial infections [18], atherosclerotic plaque, [19], and cancers [20]. This pH gradient is of particular importance since several drugs and drug carriers are taken up by endocytosis and found and/or trapped within endosomes and lysosomes. In this context, niosomes with increased affinity for an acidic pH microenvironment can take advantage of pathological conditions of inflammation for selective targeting. Recently, Rinaldi et al. [21,22] have described the formulation of pH-sensitive niosomes containing Polysorbate 20/Chems (Tween 20, Tw20) or Polysorbate 20 derivatized by Glycine (Tw20-Gly) as an effective strategy for the delivery of anaesthetics and anti-inflammatory drugs. In particular, the preparation steps (panel A) and mechanism of bilayer destabilization (panel B) are shown in Figure 1.

In this paper, the well-characterized and selected samples of the previous research study [16,17] were newly prepared to compare the analgesic activity of pH-Tw20Gly niosomes loaded with ibuprofen (NioIbu) to that of free ibuprofen in animal models of acute and chronic pain. We showed that NioIbu strongly increases Ibuprofen’s analgesic activity, promoting a longer duration of action of this drug. We suggest that NioIbu is a new and more effective strategy to treat pain arising from chronic inflammatory conditions.

## 2. Materials and Methods

### 2.1. Materials

Tween 20 (Tw20), Cholesterol >99% (Chol), *N*-(2-Hydroxyethyl)piperazine-*N*’-(2-ethanesulfonic acid) (HEPES) >99.5%, Sephadex G75, Glycine (Gly), and all other chemicals and solvents of the highest purity and of spectroscopic grade were purchased from Sigma-Aldrich (Sigma-Aldrich S.r.l., Milan, Italy). Water was purified through a Millipore Milli-Q system (Merck S.p.a., Milan, Italy).

### 2.2. Nanovesicle Formulation and Characterization

Niosomes based on a polysorbate-20 glycine derivative (Tw20Gly) were prepared by the thin film evaporation method at different Ibuprofen loading concentrations (Table 1) and purified by glass chromatography, as previously described [21,22]. A Nano ZS90 Dynamic light scattering LS (Malvern Instruments Ltd., Malvern, UK) at a scattering angle of 90.0° and a LS50B spectrofluorometer (PerkinElmer, MA, USA), were both employed for the physico-chemical characterization of vesicles (hydrodimanic diameter, size distribution, zeta potential, bilayer properties, stability, and in vitro release studies) [21]. Drug entrapment within non-ionic surfactant vesicles was determined using high-performance liquid chromatography (HPLC). HPLC analyses were carried out with a Perkin-Elmer 250 liquid chromatography apparatus (PerkinElmer, MA, USA), equipped with a Perkin-Elmer 235 photo-diode array detector, a 20-μl-loop Rheodyne injector and a computer hardware, as previously described, on purified niosomes after disruption with isopropanol (vesicle dispersion/isopropanol 1:1 *v*/*v* final ratio) [23].

Atomic force microscopy (AFM) topographical characterization has been carried out using a standard AFM setup (Dimension Icon, Bruker Inc. in ‘soft tapping’ mode, Billerica, MA, USA) equipped with standard silicon cantilevers (OTESPA, Bruker Inc., Billerica, MA, USA). Analysis of niosomal dimensions was carried out by means of atomic force microscopy (AFM) by imaging the samples in tapping mode after deposition on a Si substrate. A diameter and height of nine isolated niosomes were measured. These allowed us to determine the surface of the niosomes deposited on the substrate (which obviously lost their original spherical shape) given by the sum of areas of the surface of the spherical cap and of the base circle. Assuming that the flattening of niosomes resulted in the modification of shape and volume without significantly affecting the surface area, the diameter of niosomes in solution was evaluated as that of a sphere having the same surface area.

### 2.3. Animals and Treatments

We used male CD-1 mice (Harlan, Italy) weighing 25 g in all of the experiments. Mice were housed in colony cages under standard conditions of light, temperature, and relative humidity for at least 1 week before the start of experimental sessions. All experiments were performed according to Legislative Decree 26/14, which implements the European Directive 2010/63/UE on laboratory animal protection in Italy, and were approved by the local ethics committee. Animal studies are reported in accordance with the ARRIVE (Animal Research: Reporting of In Vivo Experiments) guidelines [24].

In all experiments, NioIbu 5%, diluted to obtain the same drug concentration, was compared to: (i) Hepes buffer (HB), (ii) an “unstructured” surfactant formulation (TG), composed of surfactant and cholesterol, and (iii) an “unstructured” surfactant formulation (TG-Ibu), composed of surfactant, cholesterol, and ibuprofen at the same concentrations as the ones present in NioIbu, but not organized in vesicular systems.

### 2.4. Writhing Test

The procedure was similar to the one previously described [25]. After 1 h of adaptation to transparent cages, mice received a subcutaneous (s.c.) injection of 1500 L of HB, TG, TG-Ibu, and NioIbu into the loose skin over the interscapular area. One hundred and twenty minutes after sample injection, mice received an intraperitoneal (i.p.) injection (10 mL/kg) of 0.6% acetic acid solution. A writhe is characterized by a wave of abdominal muscle contractions accompanied by body elongation and the extension of one or both hind limbs. The number of writhes in a 20-min period was counted, starting 5 min after acetic acid injection.

### 2.5. Capsaicin-Induced Paw Licking

The method used was similar to the one previously described [26]. Mice were allowed to adapt to transparent cages individually for 1 h before testing. Then, s.c. injections of samples (HB, TG, TG-Ibu, and NioIbu, 40 µL) were performed in the dorsal surface of mice hind paw for 120 min before 1.6 µg of capsaicin (20 µL). A micro syringe with a 26-gauge needle was used to inject capsaicin and samples. The time (in seconds) the animals spent licking the injected paw, for a total period of 5 min, was registered and considered as indicative of pain. Capsaicin was dissolved in DMSO as a stock solution and stored at −20 °C. On the test day, this solution was diluted in order to obtain the final concentration of 1.6 µg/20 µl in DMSO:saline (1:3 *v*:*v*).

### 2.6. Zymosan-Induced Hyperalgesia

In these experiments, 20 μL of zymosan A (2.5% *w*/*v* in saline) were administered s.c. into the dorsal surface of one hind paw. Then, thermal thresholds were determined, as previously reported [27]. Briefly, mice were placed in clear plastic boxes with a glass floor and allowed to acclimatize to their surroundings for at least 1 h in a temperature-controlled (21 °C) experimental room for three consecutive days prior to testing. On the test day, the animals were acclimatized to the experimental room 1 h before paw withdrawal latency (PWL) was measured. Attention was taken to start the test when the animal was not walking, with its hind paw in contact with the glass floor of the apparatus. A radiant heat source was directed at the mouse footpad until an aversive action was observed, such as paw withdrawal, foot drumming, or licking. A timer automatically measured in seconds the paw withdrawal latency. The heat source was set to an intensity of 30 and a cutoff time of 15 s was used to prevent tissue damage. Animals were first tested to determine their baseline PWL; after zymosan injection, the PWL (s) of each animal in response to the plantar test was determined again at 1, 2, 3, 4, 5, 24, and 48 h. In these experiments, HB, TG, TG-Ibu, and NioIbu were injected s.c. in a volume of 40 L in the dorsal surface of mice hind paw, 15 min before zymosan injection.

### 2.7. Neuropathy-Induced Allodynia and Hyperalgesia

The chronic constriction injury (CCI) model was carried out as previously described [28], with slight modifications [23]. Briefly, mice were anesthetized with chloralium hydrate-xylazine (400 + 10 mg/kg, i.p.). The right sciatic nerve was exposed at the mid-thigh level and, in the vicinity of the sciatic nerve trifurcation, was loosely tied with two ligatures of nylon black monofilament (9–0 non-absorbable, S&T, Neuhausen, Switzerland). Ligatures spaced 1.5–2 mm apart. Then, the muscles and the skin were closed with sutures. These animals were used in two nociceptive tests in the following order: mechanical allodynia followed by thermal hyperalgesia. The threshold for mechanical allodynia was assessed with the dynamic plantar aesthesiometer (Ugo Basile, Italy). Animals were trained on the apparatus, consisting of clear cages with a wire mesh floor, for 3 days before experimental sessions to allow for acclimatization. When the animal was at rest, a straight metal filament exerting an increasing upward force at a constant rate (5 g/s) with a maximum cutoff force of 50 g was placed under the plantar surface of the hind paw. Measurement was stopped when the paw was withdrawn and the results were expressed in grams. The measurement was repeated three times for each paw and averaged.

The development of thermal hyperalgesia was measured by subjecting the injured animals to the plantar test, as described in the “Zymosan-induced hyperalgesia” section.

Behavioural assessments of CCI-induced allodynia and thermal hyperalgesia were carried out 10 days after nerve injury, and behavioural responses compared with the ones measured before surgery. In these experiments, s.c. injection volumes of 40 L of HB, TG, TG-Ibu, and NioIbu were performed in the dorsal surface of mice hind paw.

### 2.8. Data Analysis and Statistics

Experimental data are presented as mean ± standard error of the mean (s.e.m.). Statistically significant differences between groups were calculated with an analysis of variance (ANOVA) followed by Tukey’s post-hoc comparisons. Data were analyzed using the GraphPad Prism 6.03 software. The criterion for significance was set at *P* < 0.05. The data and statistical analysis conformed to the recommendations on experimental design and analysis in pharmacology [29].

## 3. Results

### 3.1. Nanovesicle Formulation and Characterization

**Figure 2:**
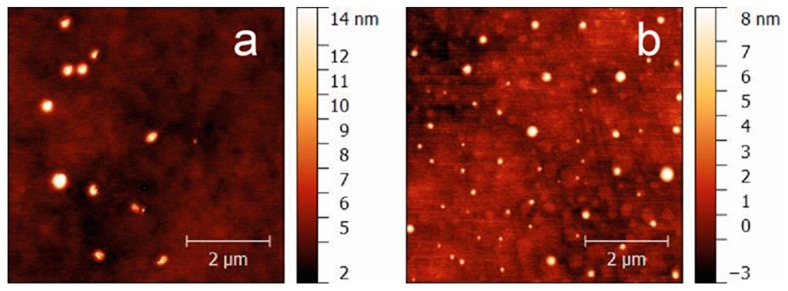
AFM images of Nio (**a**) and NioIbu (**b**) samples.

All in vivo tests were performed by administration of well-characterized (Table 2) and stable (at least 3 months when stored at 4 °C) pH-sensitive vesicles (Nio, NioIbu). AFM characterization indicates that niosomes have a spherical shape (Figure 2). In agreement with DLS, AFM images indicate that NioIbu nanovesicles are smaller and have a less-uniform size than the Nio ones. Sizes deduced from AFM images seem underestimated with respect to the DLS results. In particular, for both the samples, the diameter evaluated by AFM is 70% of that evaluated by DLS, probably due to the approximations assumed in the model. In particular, Ibuprofen-loaded vesicles show the appropriate dimensions and zeta potential to be tested in animal studies. Drug entrapment efficiency is useful to perform in vivo studies, and the bilayer fluidity is quite high to ensure drug release, as reported in Rinaldi et al. [21]. This selection was carried out based on the best in-vitro performance of the sample, in particular in terms of the percentage of released drug [21]; in this previous study, sample characterization in the presence of different pH conditions was carried out, and the pH sensitivity of the selected formulation was confirmed.

### 3.2. Writhing Test

**Figure 3:**
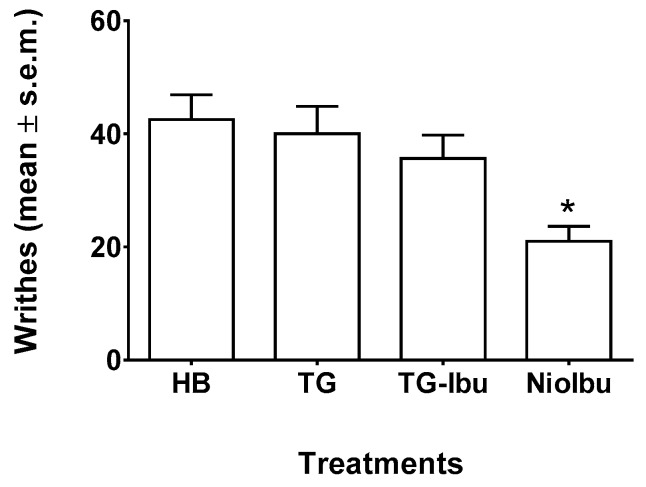
Effects of Hepes buffer (HB), the unstructured surfactant formulation (TG), the unstructured surfactant formulation with the same Ibuprofen (IBU) concentration (TG-Ibu), and TW20-Gly loaded with IBU (NioIbu) on the number of writhes induced by acetic acid. Samples were subcutaneously injected 120 min before acid acetic injection. * is for *P* < 0.05 versus TG. *N* = 8.

The in vivo antinociceptive activity of NioIbu was first assessed through a writhing test (Figure 2). As detailed in the experimental procedure section, acetic acid was used to induce peripheral pain. Analgesic activity was determined by recording the decrease in the number of writhes after acetic acid injection. The acetic-acid-induced writhing test in mice is the most commonly used method for measuring preliminary antinociceptive activity, since, in this test, both central and peripheral analgesics are detected. In these experiments, tested mice were injected with acetic acid 120 min after drug treatment. Statistical analysis revealed significant differences between treatments [*F*_(3, 28)_ = 5.545, *P* = 0.0041]. In this test, the administration of TG or TG-Ibu did not change the response to acetic acid in mice (Figure 3). Strong inhibition of the number of writhes was instead observed when NioIbu was administered 120 min before the acetic acid (Figure 3). In comparison with the TG group, NioIbu significantly reduced the number of writhes (*P* < 0.05), and the inhibition ratio was 52.5.

### 3.3. Capsaicin Test

**Figure 4:**
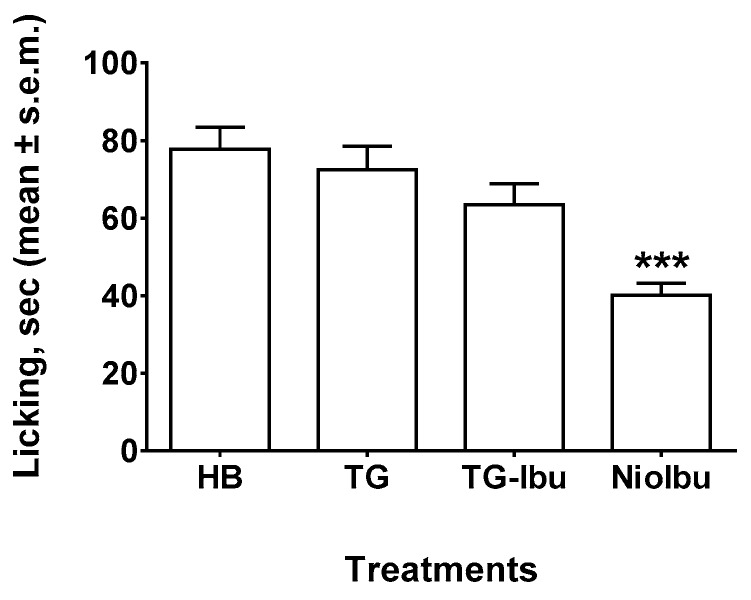
Effects of Hepes buffer (HB), the unstructured surfactant formulation (TG), the unstructured surfactant formulation with the same Ibuprofen (IBU) concentration (TG-Ibu), and TW20-Gly loaded with IBU (NioIbu) on the time spent licking the capsaicin-injected paw. Samples were injected subcutaneously in the dorsal surface of the hind paw 120 min before capsaicin injection. *** is for *P* < 0.001 versus TG. *N* = 10.

In order to better evaluate the antinociceptive activity of ibuprofen-loaded vesicles, we also performed the capsaicin test (Figure 4). This test reflects acute pain responses related to neurogenic inflammation. In sensory neurons, capsaicin is an activator of the TRPV1 channels present in C-fibers and, to a lesser extent, Aδ. After injection, capsaicin shows a biphasic effect, i.e., it stimulates TRPV1 located in sensory neurons, producing a rapid phase of burning sensation, and local vascular and extravascular responses, followed by a persistent desensitization with concomitant long-lasting analgesia. Statistical analysis revealed significant differences between treatments, as revealed by ANOVA (*F*_(3, 36)_ = 10.22, *P* < 0.0001). In this test, neither TG nor TG-Ibu reduced the duration of the licking response as compared with the HB-treated mice (Figure 4). A statistically significant antinociceptive effect was shown for NioIbu (inhibition ratio, 55.4%, *P* < 0.001) versus TG-treated mice.

### 3.4. Zymosan-Induced Hyperalgesia

**Figure 5:**
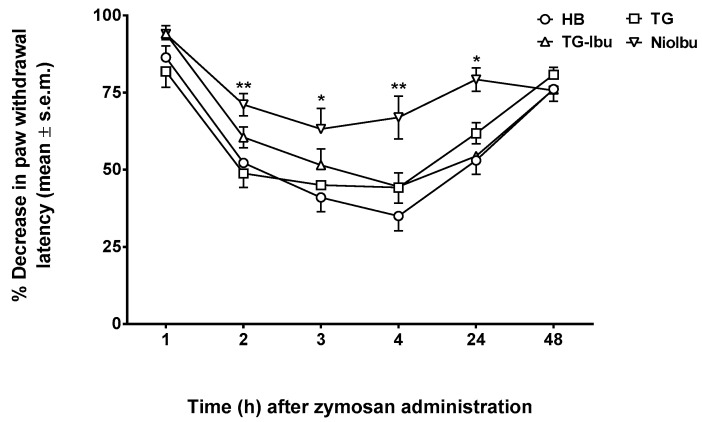
Effects of Hepes buffer (HB), the unstructured surfactant formulation (TG), the unstructured surfactant formulation with the same Ibuprofen (IBU) concentration (TG-Ibu), and TW20-Gly loaded with IBU (NioIbu) on zymosan-induced hyperalgesia. * is for *P* < 0.05 and ** is for *P* < 0.01 versus TG. *N* = 10.

A s.c. injection of zymosan into mice footpad induces persistent dose- and time-dependent thermal and mechanical hyperalgesia associated with inflammation up to 24 h after treatment (Figure 5). Primary hyperalgesia in the hind paw inflammation model is thought to result from a release of pro-inflammatory mediators that include bradykinin, cytokines, and prostaglandins [30,31]. The reduction in latency response to a thermal stimulus applied to a paw and induced by zymosan was measured as a percentage. In these experiments, samples were injected into the dorsal surface of the right hind paw 15 min before zymonsan injection. As observed in the writhing and capsaicin test, TG and TG-Ibu did not affect the decrease in nociceptive threshold induced by zymosan. On the contrary, the highest increase in pain threshold was observed in NioIbu-treated mice. (Figure 5). Furthermore, the increase in the nociceptive threshold induced by NioIbu was long-lasting, since two-way ANOVA revealed significant differences from 3 to 24 h after NioIbu treatment (*F*_(10, 135)_ = 3.44, *P* = 0.0005).

### 3.5. Neuropathy-Induced Allodynia and Hyperalgesia

**Figure 6:**
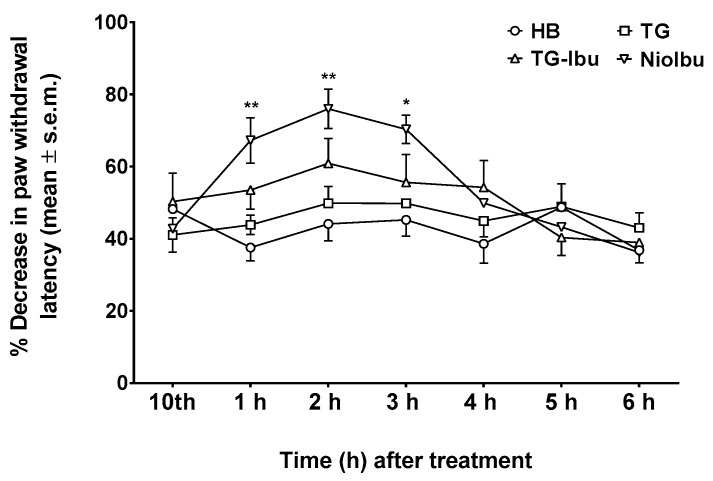
Effects of Hepes buffer (HB), the unstructured surfactant formulation (TG), the unstructured surfactant formulation with the same Ibuprofen (IBU) concentration (TG-Ibu), and TW20-Gly loaded with IBU (NioIbu) on allodynia induced by a chronic constriction injury of the right sciatic nerve. * is for *P* < 0.05 and ** is for *P* < 0.01 versus TG. *N* = 8.

**Figure 7:**
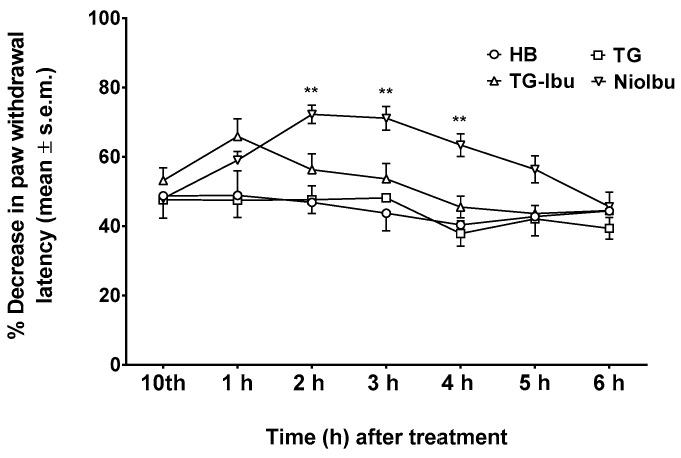
Effects of Hepes buffer (HB), the unstructured surfactant formulation (TG), the unstructured surfactant formulation with the same Ibuprofen (IBU) concentration (TG-Ibu), and TW20-Gly loaded with IBU (NioIbu) on hyperalgesia induced by a chronic constriction injury of the right sciatic nerve. * is for *P* < 0.05 and ** is for *P* < 0.01 versus TG. *N* = 8.

Neuropathic pain is a chronic condition caused by injury to the nervous system. This condition is characterized by spontaneous pain, as well as by exaggerated pain responses to painful stimuli (hyperalgesia) and to normally non-painful stimuli (allodynia). In our experiments, allodynia and hyperalgesia were measured 10 days after nerve injury, because this is when the pain threshold reaches the minimum value. The results obtained in these experiments are shown in Figure 6 and Figure 7. When allodynia was measured (Figure 6), two-way ANOVA showed a significant difference between treatments (*F*_(12, 126)_ = 4.453; *P* < 0.0001). A Tukey’s multiple comparison test demonstrated that NioIbu significantly increased paw withdrawal latency from 1 to 3 h after treatment, whereas TG-Ibu induced a transient, not significant increase in the nociceptive threshold (Figure 6).

In the same way, two-way ANOVA revealed significant differences in pain threshold when hyperalgesia was measured (*F*_(12, 126)_ = 6.811; *P* < 0.0001) (Figure 7). NioIbu increased the pain threshold from 2 h up to 4 h after treatment, whereas the increase in the pain threshold observed 1 h after TG-Ibu administration was not statistically significant.

## 4. Discussion

Several researchers have focused on alternative drug delivery systems to overcome the difficulties associated with the distribution and effectiveness of analgesic drugs. This is mainly due to the low capability of analgesic drugs to pass across biological membranes [32]. One of the strategies adopted to overcome these limitations was loading the drugs into liposomes, which are spherical vesicles composed of phospholipids, such as phosphatidylcholine and phosphatidyl-serine, and possibly other lipids as well [33]. However, liposomes have high production costs due to the phospholipids employed and tend to fuse or aggregate, resulting in an early release of the vesicle payload. Moreover, phospholipids are prone to oxidative degradation. Finally, liposomes have low solubility and stability, and their applicability is limited because they have short half–lives in blood circulation [13,34,35]. To overcome these constraints, we decided to use a new non-toxic drug delivery system, the niosomes. These carriers offer several advantages compared to liposomes: a higher capability to entrap lipophilic, hydrophilic, and amphiphilic drugs; an ability to reach the site of action via oral, parenteral, and topical routes of administration; and a lower cost. [36]. Recently, Di Marzio et al. [13] described specific pH-sensitive, non-ionic surfactant vesicles with polysorbate-20 (Tween-20)/Chems as a good delivery system for analgesic drugs, which increase the stability and affinity in the tissue pH alterations that occur during inflammatory pathologic conditions [37]. These vesicles showed the ability to control and sustain release as well as to protect drugs from catalytic enzymes, thereby increasing drug stability.

Niosomes containing pH-sensitive components, such as TW20Gly, that protonate at lower pH [35] showed bilayer destabilization, leading to localized drug release. This approach could be useful in targeting inflamed tissues. In this study, we evaluated the antinociceptive effects of s.c. injections of Ibuprofen-loaded, pH-sensitive niosomes in comparison with the free form of the drug. To address this issue, we utilized mice under different pain stimuli. This work demonstrates that the use of these nanocarriers enhances the therapeutic efficacy of Ibuprofen, since Ibuprofen-loaded niosomes were effective in reducing pain in laboratory mice. The use of nanovesicles as delivery systems has already been successfully exploited to supply compounds topically and orally. In fact, some studies showed that NSAID-loaded vesicles have better performance in terms of prolonged drug release [38] and improved therapeutic effects [39] compared with the free form of the same compound. Considering that niosomes are a very promising system to administer compounds of different features topically or orally [40,41,42], we evaluated the capability of niosomes to increase antinociceptive effects of Ibuprofen also in terms of lasting effects after s.c injection. Writhing and capsaicin tests are screening assays commonly used to study peripheral and central antinociceptive activity. Our results showed that, when Ibuprofen-loaded niosomes were administered, there was an increase in the positive response time. In fact, when Ibuprofen was given 120 min earlier, the antinociceptive activity was maintained only in the niosomal form and not in the free form. The capsaicin test confirmed the long-lasting effects of Ibuprofen-loaded niosomes. Similar results were obtained with diclofenac-loaded liposomes administered orally, as reported by Goh et al. [43]. Consistently, in a formalin test, Ibuprofen-loaded niosomes displayed a peripheral antinociceptive activity and were more effective than free Ibuprofen in suppressing inflammatory pain. The niosomal form also had a long-lasting action, while the free form was probably rapidly catabolized [16]. Therefore, the encapsulation of the drug into niosomes significantly enhances drug efficacy and increases the durability of antinociceptive effects. This allows for a reduction in doses and, consequently, might also reduce the drug’s side effects.

Some studies reported that nano-formulations reduce hyperalgesic responses to thermal stimuli more efficiently than free drugs [44,45]. Our results on zymosan-induced hyperalgesia are in line with these studies and demonstrate that Ibuprofen-loaded, pH-sensitive niosomes were able to reduce nociception up to 24 h after administration. These results confirm that niosomes release the encapsulated drug efficiently when behavioural experiments were performed. Similarly, Ibuprofen-loaded niosomes decrease neuropathic pain, since the treatment reduced both hyperalgesia and allodynia induced by chronic sciatic nerve constriction.

The increase in drug activity when released by niosomes could be explained by the capability of niosomes to transport lipophilic drugs, in this case Ibuprofen, across tissue membranes. This facilitates drug diffusion around the site of application and consequently improves efficacy [13,22]. 

In conclusion, the present study reveals that Ibuprofen-loaded, pH-sensitive niosomes are able to produce consistent and long-lasting antinociceptive effects. The antinociceptive effects of Ibuprofen-loaded niosomes were observed both in acute and chronic animal models of pain and these results, together with those reported in our previous study [21], suggest that an Ibuprofen pH-sensitive nanocarrier formulation could be further developed and used to treat different pain conditions in humans.

## Figures and Tables

**Table 1:** Sample composition (NioIbu 5% has been the analyzed formulation). Nio, pH-Tw20Gly niosomes; NioIbu, pH-Tw20Gly niosomes loaded with different percentages of Ibuprofen Hepes solutions. Tw 20, is for Tween 20; Chol, is for Cholesterol; IBU, is for Ibuprofen.

Sample	Tw20 (mM)	Tw20-Gly (mM)	Chol (mM)	IBU (% *p*/*v*)
Nio	3.75	11.25	7.5	=
NioIbu 1%	3.75	11.25	7.5	1
NioIbu 3%	3.75	11.25	7.5	3
NioIbu 5%	3.75	11.25	7.5	5
NioIbu 7%	3.75	11.25	7.5	7

**Table 2:** Vesicle characterization. Nio, pH-Tw20Gly niosomes; NioIbu, pH-Tw20Gly niosomes loaded with a 5% Ibuprofen Hepes solution.

Niosomes	Diameter (nm)	AFM Diameter (nm)	ζ Potential (mV)	Polydispersity Index	Fluorescence Anisotropy (AU)	Loaded Drug Conc. (mg/mL)
Nio	215.0 ± 3.0	152 ± 18	−41.0 ± 1.2	0.160 ± 0.08	0.17 ± 0.01	–
NioIbu 5%	122.1 ± 19.6	89 ± 24	−40.2 ± 0.1	0.404 ± 0.05	0.20 ± 0.04	0.37 ± 0.05
